# Japan Coma Scale used in the prehospital setting can predict clinical outcome in severe pediatric trauma

**DOI:** 10.1186/cc12262

**Published:** 2013-03-19

**Authors:** T Yagi, N Saito, Y Hara, H Hisashi Matumoto, K Mashiko

**Affiliations:** 1 Nippon Medical School Chiba Hokusoh Hospital, Inzai/Chiba, Japan

## Introduction

In the prehospital setting, it is difficult to use the Glasgow Coma Scale (GCS) to evaluate the consciousness state using in pediatric patients with severe trauma. The Japan Coma Scale (JCS) is a consciousness scale used widely in Japan and, with its four grades, is simpler and quicker to use than the GCS. This study examined whether the JCS can predict clinical traumatic brain injury (TBI) and outcome in pediatric patients aged 3 to 15 years in the prehospital setting.

## Methods

This retrospective study analyzed data from the Japan Trauma Data Bank. Registered pediatric patients aged 3 to 15 years with severe trauma (maximum Abbreviated Injury Scale score ≥3 or Injury Severity Score ≥9) were divided into four groups according to JCS score in the prehospital setting (Grade 0: alert, Grade 1: possible eye-opening, not lucid, Grade 2: possible eye-opening upon stimulation, Grade 3: no eye-opening and coma). TBI was defined as maximum head AIS ≥3.

## Results

A total of 1,562 patients were included (Grade 0: 673, Grade 1: 410, Grade 2: 230, Grade 3: 249). Victims of blunt trauma accounted for 98.1%. Median age was 9 (interquartile range: 7 to 12) years, median ISS was 16 (9 to 21). There was strong agreement between the JCS in prehospital setting and GCS scores on arrival at hospital (*r *= -0.745, *P <*0.001). Multivariate analysis adjusted for age and ISS revealed that the JCS was independently associated with TBI (odds ratio (OR): 2.5; 95% CI: 2.1 to 2.8, *P <*0.001) and hospital mortality (OR: 3.8; 95% CI 2.4 to 6.0, *P <*0.001). See Table 1.

## Conclusion

There was strong association between JCS score and clinical outcome in pediatric patients with severe trauma. The results support the use of the JCS in the prehospital transport destination criteria for children.

**Table 1:** 

	TBI, *n *(%)	Mortality (%)
JCS grade 0	213 (31.6)	0.1
JCS grade 1	225 (62.2)	0.7
JCS grade 2	184 (80.0)	2.6
JCS grade 3	230 (92.4)	15.7

